# Telemedicine Use During the COVID-19 Pandemic in 8 Countries From the International Sexual Health and Reproductive Health Consortium: Web-Based Cross-Sectional Survey Study

**DOI:** 10.2196/60369

**Published:** 2025-03-04

**Authors:** Rayner Kay Jin Tan, Devon Hensel, Olena Ivanova, Raquel Gomez Bravo, Adesola Olumide, Emmanuel Adebayo, Amanda Cleeve, Amanda Gesselman, Sonam Jyoti Shah, Helen Adesoba, Gifty Marley, Weiming Tang

**Affiliations:** 1 Saw Swee Hock School of Public Health National University of Singapore and National University Health System Singapore Singapore; 2 University of North Carolina Project-China Guangzhou China; 3 Department of Pediatrics Indiana University School of Medicine Indianapolis, IN United States; 4 Department of Sociology Indiana University School of Medicine Indianapolis, IN United States; 5 Institute of Infectious Diseases and Tropical Medicine LMU University Hospital LMU Munich Munich Germany; 6 German Center for Infection Research (DZIF) Partner Site Munich Munich Germany; 7 Department of Behavioural and Cognitive Sciences University of Luxembourg—Campus Belval Belval Luxembourg; 8 Institute of Child Health, College of Medicine University of Ibadan and University College Hospital Ibadan Nigeria; 9 Department of Global Health Karolinska Institutet Stockholm Sweden; 10 Department of Women's and Children's Health Karolinska Institutet Stockholm Sweden; 11 Kinsey Institute Indiana University Bloomington, IN United States; 12 Institute for Global Health and Infectious Diseases University of North Carolina at Chapel Hill Chapel Hill, NC United States; 13 School of Medicine University of North Carolina at Chapel Hill Chapel Hill, NC United States; 14 Dermatology Hospital of Southern Medical University Guangzhou China

**Keywords:** COVID-19, telemedicine, sexual and reproductive health, pandemic, web-based survey, sexual health, reproductive health, communication technology, medical education, contraception, abortion, health care delivery, care, chronic condition

## Abstract

**Background:**

Telemedicine is an important way to fill in the access gap to in-person health care services during challenging times like pandemics.

**Objective:**

This study aimed to investigate the role that telemedicine played during the COVID-19 pandemic by multicountry comparison of the use of telemedicine prior to and during the pandemic.

**Methods:**

This study analyzes data from the second wave of the International Sexual Health and Reproductive Health study. This included data collected between April 2021 and July 2022 in 8 countries, including Armenia (n=296), Egypt (n=889), Germany (n=138), Moldova (n=311), Nigeria (n=205), Portugal (n=951), Singapore (n=13), and Spain (n=54). This study covered sociodemographics, sexual and reproductive health (SRH), and telemedicine use. Descriptive statistics and multilevel modeling were used to assess the factors influencing the use of telemedicine.

**Results:**

Overall, 2857 participants were recruited. Approximately 57.6% (n=1646) of participants had never used telemedicine prior to COVID-19 measures, while 45.9% (n=1311) of participants required health care but reported not using telemedicine services following the introduction of COVID-19 measures. In high-income countries, the most common mode reported was audio-based telemedicine services, with 283 (71.8%) and 417 (73.5%) participants doing so before and during COVID-19, respectively. This was followed by text-based telemedicine services, with 152 (38.6%) and 173 (30.5%) participants doing so before and during COVID-19, respectively. In low- to middle-income countries, many participants also reported using audio-based telemedicine services, with 288 (35.3%) and 237 (40.8%) participants doing so before and during COVID-19, respectively. This was followed by chat-based telemedicine services, with 265 (32.4%) and 217 (37.3%) participants doing so before and during COVID-19, respectively. Multilevel modeling revealed that those who were older (adjusted odds ratio [aOR] 0.99, 95% CI 0.99-1.00) and were in countries with a higher gross domestic product per capita (aOR 0.99, 95% CI 0.98-1.00) were less likely to have ever used telemedicine. Participants who were of male sex assigned at birth (aOR 0.79, 95% CI 0.65-0.96) were less likely to use telemedicine during the pandemic. Participants who perceived that they were worse off financially were more likely to have switched to telemedicine during COVID-19 (aOR 1.39, 95% CI 1.02-1.89) and were more likely to report having a poor or fair experience of telemedicine services (aOR 1.75, 95% CI 1.34-2.29). When sexual orientation was included in the model, nonheterosexual individuals were more likely to ever use telemedicine prior to COVID-19 (aOR 1.35, 95% CI 1.08-1.69), more likely to have used telemedicine during COVID-19 (aOR 1.58, 95% CI 1.24-2.02), and more likely to have switched to telemedicine during COVID-19 (aOR 1.55, 95% CI 1.09-2.21).

**Conclusions:**

Telemedicine played a key role in addressing health care needs during the COVID-19 pandemic. Age, sex, economic status, and sexual orientation influenced its use.

## Introduction

The World Health Organization defines telemedicine as “healing at a distance,” enabling the use of information and communication technology tools to improve the quality of medical services while overcoming the obstacles posed by travel [1]. It has also been described as “a branch of e-health that uses communication networks for the delivery of healthcare services and medical education from one geographic location to another” [2]. Telemedicine plays an important role in enhancing or substituting access to health care services, particularly during challenging circumstances like a pandemic or in hard-to-reach areas [3]. Moreover, telemedicine technologies have the potential to address unmet sexual and reproductive health (SRH) needs and improve SRH services. This is especially important as the World Health Organization estimates that everyone of reproductive age would not have access to at least 1 essential reproductive health intervention over the course of their lives [4].

The role of telemedicine in health care has grown rapidly and concomitantly with the expansion of communication technology. The significance of telemedicine was especially underscored during COVID-19 infection control measures and lockdowns. During this time, telemedicine provided the general public with a convenient and secure method to seek guidance from health care professionals regarding COVID-19 symptoms, preventive and treatment measures, psychological concerns, and various other matters [5]. It is estimated that about 116 million people had access to digital doctor consultations worldwide in 2024 compared to 57 million in 2019 [6]. During the pandemic, telemedicine was also recommended to be used for contraception counseling, shared decision-making, and managing potential side effects [7]. It was also used as a means of accessing abortion-related services during the pandemic [8]. Country-specific studies demonstrate that telemedicine was successfully implemented to provide SRH services during the pandemic, and it was a convenient and comfortable approach, especially for young people [9,10].

Telemedicine helped fill several health care service gaps that emerged during the pandemic, especially in the context of SRH [11]. First, telemedicine allowed patients to consult with health care providers remotely, reducing the need for in-person visits to health care facilities [12]. This was particularly important during the pandemic when minimizing physical contact was essential to curb the spread of the virus. Second, telemedicine platforms were used for COVID-19 screening and triage [5]. Patients could use digital visits to discuss symptoms with health care professionals, who could then determine the appropriate course of action, whether it be self-isolation, testing, or in-person care. Third, many routine and nonemergency health care services were disrupted due to lockdowns and restrictions [13,14]. Telemedicine provided a way for patients to continue receiving medical care for chronic conditions, follow-up appointments, medication management, and health services without needing to visit a health care facility. Fourth, the pandemic had a significant impact on mental health, and telemedicine became a valuable tool for delivering mental health services [15]. Digital counseling and therapy sessions allowed individuals to access support from the safety of their homes.

In the past 2021-2022 period, many countries have relaxed COVID-19 restrictions. This provided a unique opportunity to understand how social and behavioral factors related to decreasing COVID-19 restrictions may have influenced sexual behaviors in diverse settings, including the use of telemedicine. The second International Sexual Health and Reproductive Health (I-SHARE)-2 study examined the 2021-2022 period and focused on similar sexual or reproductive health data from I-SHARE-1, over and above the addition of telemedicine-related variables. The aim of this study is to describe the use of telemedicine before and during the COVID-19 pandemic in 8 countries. The data for this study are a part of the cross-sectional multicountry project called “International Sexual Health and Reproductive Health during COVID-19” [16]. The I-SHARE project brought together SRH researchers from different countries to conduct a web-based survey using a standardized instrument. The main aim of this multinational study was to gain insights into SRH before and during the initial phase of the COVID-19 pandemic in each country.

The I-SHARE, multicountry, web-based, cross-sectional study was developed to assess SRH during and after COVID-19 restrictions. The initial multicountry study examined the 2020-2021 period and compared SRH in the pre–COVID-19 and COVID-19 periods [17,18]. In this study, we found that the pandemic hindered access to SRH care. However, telemedicine-related variables were not measured in the initial study. This study aimed to understand the prevalence of general telemedicine use and correlates of its use during the COVID-19 period as well as prior to COVID-19.

## Methods

### Study Design and Participants

This study analyzes data from the I-SHARE study. The consortium was developed to better understand SRH during the COVID-19 pandemic. The initial round of data collection (named the “I-SHARE survey”) covered the period from July 20, 2020, to February 15, 2021. This I-SHARE-2 survey included data collected between April 2021 and July 2022 in 8 countries, including Armenia (n=296), Egypt (n=889), Germany (n=138), Moldova (n=311), Nigeria (n=205), Portugal (n=951), Singapore (n=13), and Spain (n=54).

Each country included details of SRH resources at the end of the survey. The in-country team translated the survey into local languages, field-tested the survey instrument, and submitted the protocol to a local institutional review committee for review. Field testing included giving the survey to at least 10 individuals who provided feedback about translation and sensitive topics. Some countries organized a second round of field testing using the web-based version of the survey questionnaire. All countries recruited participants on the web and through the help of community organizations. More details of the methods of the initial I-SHARE survey are described in the survey protocol [19]. The survey took approximately 15-20 minutes to complete. Open Data Kit software (version 1.16; Get ODK Inc) was used to collect data from participants on a cell phone, laptop, or other electronic device.

Eligibility criteria for participation included being at least 18 years of age, residing in the country where the survey was conducted, and being able to provide web-based informed consent. Researchers from each country were invited to join working groups focused on analyzing multicountry data. Survey data were included if they had received institutional review board approval and field-tested the survey. The survey questionnaire included domains on sociodemographic characteristics, sexual relationships, compliance with COVID-19 restrictions (eg, social distancing), web-based harassment through digital media, intimate partner violence, and HIV or sexually transmitted disease testing. The complete survey instrument is included in Table S1 in Multimedia Appendix 1.

### Measures

Telemedicine use was measured through a series of questions regarding its (1) use prior to the introduction of COVID-19 measures (never, rarely, sometimes, often, and always), (2) use following the introduction of COVID-19 measures (yes, no, and did not need to access health care), (3) the various types of telemedicine modalities used (audio, video, text, chat, or other forms), as well as (4) participants’ satisfaction with telemedicine services during COVID-19 (excellent, good, fair, and poor). While this was a survey that focused on SRH, our questions on telemedicine use were not limited to just SRH services, and we were not able to measure the use of telemedicine in the context of specific types of services. We constructed a new variable for participants who never used telemedicine prior to the introduction of COVID-19 measures but needed health care and switched to the use of telemedicine following the introduction of COVID-19 measures (switched to telemedicine).

Individual-level variables included sociodemographic characteristics such as sex assigned at birth, age (in years), sexual orientation, place of residence, educational attainment, employment status, partner living arrangement, perceived changes to one’s economic situation as a result of COVID-19, and having children at home. Country-level variables included gross domestic product (GDP) per capita.

### Data Analysis

Sociodemographic characteristics as well as frequencies and proportions around the use of telemedicine were summarized using descriptive statistics. Subsequently, we proceeded to conduct multilevel modeling (using Stata, version 16; StataCorp LLC), which included country-level variables. These multilevel models were constructed to assess the odds of ever using telemedicine, of using telemedicine during COVID-19 if health care services were needed, of switching to telemedicine during COVID-19, as well as having fair or poor experiences of telemedicine during COVID-19. We omitted sexual orientation from the main models, as participants in several countries largely omitted responding to this question but provided supplemental analyses including sexual orientation as a variable. Statistical significance was set at *P*<.05.

### Ethical Considerations

Each country’s ethical review committee approved the respective national web-based study prior to launch. We obtained approval from Ghent University (approval BC-07988) and the University of North Carolina at Chapel Hill (approval 295989) for multicountry analyses based on deidentified data, which allowed for secondary analyses without additional consent. A single data-sharing agreement covered multicountry analyses. In-country leads made final decisions about data sharing and data management practices. Survey participants were required to provide informed consent prior to the survey and were allowed to stop at any point and leave any item blank. We did not collect any personal identifiers and therefore all responses were anonymous. In-country leads also made decisions on the forms of remuneration provided to participants that were appropriate for the respective countries and approved by the local ethics board.

## Results

A total of 2857 participants were recruited in the 8 countries of our sample. Most participants identified as cisgender female (n=1757, 61.5%), were heterosexual or straight (n=1313, 70.8%), reported living in the city (n=1392, 49.7%), had completed college (n=1421, 51%), were formally employed (n=1663, 58.2%), were not partnered or not living with their partners (n=1714, 60%), perceived that the economic situation of their household had remained the same (n=1400, 50.6%), and did not have children (n=1538, 60.1%). The average age of participants was 37.4 (SD 16.0) years, and the average GDP per capita at the country level was US $13,900 (SD US $13,600). Table 1 demonstrates the sociodemographic attributes of the study sample.

**Table 1:** Sociodemographic attributes of International Sexual Health and Reproductive Health-2 study participants in 8 countries, April 2021 to July 2022 (N=2857).

Demographic variables		Values
**Sex, n (%)**		
	Female	1757 (61.5)
	Male	1088 (38.1)
	Other	12 (0.4)
	Total	2857 (100)
Age (years), mean (SD)		37.4 (16)
**Sexual orientation, n (%)**		
	Heterosexual or straight	1313 (70.8)
	Minority sexual orientation	542 (29.2)
	Total	1855 (100)
**Area, n (%)**		
	City	1392 (49.7)
	Suburb of city	312 (11.1)
	Town	640 (22.8)
	Remote or rural area	420 (15)
	Other	39 (1.4)
	Total	2806 (100)
**Schooling, n (%)**		
	Secondary school and below	626 (22.4)
	Some college	609 (21.8)
	Completed college	1421 (51)
	Other	134 (4.8)
	Total	2793 (100)
**Employment status, n (%)**		
	Formally employed	1663 (58.2)
	Not formally employed	1194 (41.8)
	Total	2857 (100)
**Relationship (participants can select multiple options; denominator is n=2857), n (%)**		
	Not partnered or not living with a partner (single or partnered but not living or separated)	1714 (60)
	Partnered, living with partner	1143 (40)
	Total	2857 (100)
**Economic situation, n (%)**		
	Economic situation of household became worse	1155 (41.8)
	Economic situation of household remained the same	1400 (50.6)
	Economic situation of household became better	211 (7.6)
	Total	2769 (100)
**Has children, n (%)**		
	No	1538 (60.1)
	Yes	1023 (39.9)
	Total	2546 (100)
Gross domestic product per capita (US $), mean (SD)		13,900 (13,600)
**Country income group, n (%)**		
	High-income country (Germany, Portugal, Singapore, and Spain)	1156 (40.5)
	Low- to middle-income country (Armenia, Egypt, Moldova, and Nigeria)	1701 (59.5)
	Total	2857 (100)

We have found that approximately 57.6% (n=1646) of participants had never used telemedicine before the introduction of COVID-19 measures, while 45.9% (n=1311) of participants required health care but reported not using telemedicine services following the introduction of COVID-19 measures. A total of 370 (26.3%) participants who never used telemedicine services prior to the introduction of COVID-19 measures, and required health care services during the pandemic, switched to using telemedicine during the pandemic. A total of 482 participants (33.3%) reported a fair or poor experience with using telemedicine services during COVID-19. Table 2 summarizes the frequencies and proportions for variables regarding telemedicine use before and following the implementation of COVID-19 measures as well as for participants who switched or did not switch to telemedicine use during COVID-19.

**Table 2:** Use of telemedicine among International Sexual Health and Reproductive Health-2 study participants in 8 countries, April 2021 to July 2022 (N=2857).

Telemedicine variables			Values, n (%)
**Telemedicine use before the introduction of COVID-19 measures**			
	Never	1646 (57.6)	
	Rarely	519 (18.2)	
	Sometimes	496 (17.4)	
	Often	144 (5)	
	Always	52 (1.8)	
	Total	2857 (100)	
**Telemedicine use following the introduction of COVID-19 measures**			
	Yes	1148 (40.2)	
	No	1311 (45.9)	
	Did not need access to health care	398 (13.9)	
	Total	2459 (100)	
**Switched from not using telemedicine before to using telemedicine during COVID-19**			
	Yes	370 (26.3)	
	No	1036 (73.7)	
	Total	1406 (100)	
**Satisfaction with telemedicine services during COVID-19**			
	Excellent	316 (21.8)	
	Good	650 (44.9)	
	Fair	403 (27.8)	
	Poor	79 (5.5)	
	Total	1448 (100)	

Table 3 provides a summary of the proportions of participants by country who reported ever using telemedicine services before COVID-19 as well as telemedicine use following the introduction of COVID-19 measures (excluding participants who did not require health care services during this time). In high-income countries, a majority of participants reported using audio-based telemedicine services, with 283 (71.8%) and 417 (73.5%) participants doing so before and during COVID-19, respectively. This was followed by text-based telemedicine services, with 152 (38.6%) and 173 (30.5%) participants doing so before and during COVID-19, respectively. In low- to middle-income countries, many participants also reported using audio-based telemedicine services, with 288 (35.3%) and 237 (40.8%) participants doing so before and during COVID-19, respectively. This was followed by chat-based telemedicine services, with 265 (32.4%) and 217 (37.3%) participants doing so before and during COVID-19, respectively, as well as text-based telemedicine services, with 265 (32.4%) and 217 (37.3%) participants doing so before and during COVID-19, respectively. Table 4 summarizes the frequencies and proportions of participants who reported using either audio, video, text, chat, or other forms of telemedicine modalities during COVID-19. Country-level data may be found in Figures S1 and S2 in Multimedia Appendix 1.

**Table 3:** Telemedicine use before and during COVID-19 measures by country among International Sexual Health and Reproductive Health-2 study participants in 8 countries, April 2021 to July 2022 (N=2857).

Country and time frame		Used telemedicine, n (%)	Did not use telemedicine, n (%)
**Armenia**			
	Before COVID-19 (n=296)	168 (56.8)	128 (43.2)
	During COVID-19 (n=251)	133 (53)	118 (47)
**Egypt**			
	Before COVID-19 (n=889)	431 (48.5)	458 (51.5)
	During COVID-19 (n=723)	261 (36.1)	462 (63.9)
**Germany**			
	Before COVID-19 (n=138)	48 (34.8)	90 (65.2)
	During COVID-19 (n=117)	40 (34.2)	77 (65.8)
**Moldova**			
	Before COVID-19 (n=311)	136 (43.7)	175 (56.3)
	During COVID-19 (n=280)	127 (45.4)	153 (54.6)
**Nigeria**			
	Before COVID-19 (n=205)	82 (40)	123 (60)
	During COVID-19 (n=173)	60 (34.7)	113 (65.3)
**Portugal**			
	Before COVID-19 (n=951)	325 (34.2)	626 (65.8)
	During COVID-19 (n=853)	485 (56.9)	368 (43.1)
**Singapore**			
	Before COVID-19 (n=13)	3 (23.1)	10 (76.9)
	During COVID-19 (n=12)	5 (41.7)	7 (58.3)
**Spain**			
	Before COVID-19 (n=54)	18 (33.3)	36 (66.7)
	During COVID-19 (n=50)	37 (74)	13 (26)

**Table 4:** Telemedicine modalities used before and during COVID-19 measures by country income groupings among International Sexual Health and Reproductive Health-2 study participants in 8 countries, April 2021 to July 2022 (N=2857).

Types of telemedicine modalities used	High-income countries^a^		Low- to middle-income countries^b^			All countries^c^	
	Before COVID-19, n (%)	During COVID-19, n (%)	Before COVID-19, n (%)	During COVID-19, n (%)	Before COVID-19, n (%)		During COVID-19, n (%)
Audio	283 (71.8)	417 (73.5)	288 (35.3)	237 (40.8)	571 (47.2)		654 (57)
Video	36 (9.1)	116 (20.5)	113 (13.8)	81 (13.9)	149 (12.3)		197 (17.2)
Text	152 (38.6)	173 (30.5)	259 (31.7)	211 (36.3)	411 (33.9)		384 (33.4)
Chat	44 (11.2)	50 (8.8)	265 (32.4)	217 (37.3)	309 (25.5)		267 (23.3)
Other	68 (17.3)	84 (14.8)	58 (7.1)	36 (6.2)	126 (10.4)		120 (10.5)

^a^A total of 394 participants used telemedicine before COVID-19, and 567 participants used telemedicine during COVID-19. Countries included Germany, Portugal, Singapore, and Spain.

^b^A total of 817 participants used telemedicine before COVID-19, and 581 participants used telemedicine during COVID-19. Countries included Armenia, Egypt, Moldova, and Nigeria.

^c^A total of 1211 participants used telemedicine before COVID-19, and 1148 participants used telemedicine during COVID-19.

In Table 5, we have summarized the binomial logistic multilevel model assessing the odds of ever using telemedicine, of using telemedicine during COVID-19 if health care services were needed, of switching to telemedicine during COVID-19, as well as having fair or poor experiences of telemedicine during COVID-19. First, for individuals who ever used telemedicine prior to COVID-19, those who were older (adjusted odds ratio [aOR] 0.99, 95% CI 0.99-1.00) and were in countries with a higher GDP per capita (aOR 0.99, 95% CI 0.98-1.00) were less likely to have ever done so. Second, for telemedicine use during COVID-19, those who were of male sex assigned at birth (aOR 0.79, 95% CI 0.65-0.96) were less likely to use telemedicine.

**Table 5:** Correlates for telemedicine variables among International Sexual Health and Reproductive Health-2 study participants in 8 countries, April 2021 to July 2022 (N=2857).

				Telemedicine variables					
				Telemedicine use before COVID-19 (n=2485), aOR^a^ (95% CI)	Telemedicine use during COVID-19 (n=2147), aOR (95% CI)		Switch to telemedicine during COVID-19 (n=1259), aOR (95% CI)		Poor experience with telemedicine during COVID-19 (n=1271), aOR (95% CI)
**Variables for main multivariable analysis**									
	**Sex assigned at birth (reference=female)**								
		Male	1.16 (1.16-1.39)		*0.79* ^b^ *(0.65-0.96)* ^c^	0.77 (0.56-1.06)		0.92 (0.69-1.23)	
		Other	8.22 (8.23-70.03)		4.91 (0.53-45.27)	—^d^		1.69 (0.31-9.11)	
	Age (years)			*0.99* ^b^ *(0.99-1.00)*	0.99 (0.98-1.01)		1 (0.99-1.02)		1 (0.99-1.02)
	**Place of residence (reference=city)**								
		Suburb	1.12 (1.12-1.47)		1.19 (0.89-1.6)	1.1 (0.72-1.69)		1.28 (0.87-1.87)	
		Town	1.06 (1.06-1.32)		8.42 (0.66-1.07)	0.97 (0.66-1.42)		0.95 (0.68-1.33)	
		Remote or rural area	0.85 (0.85-1.1)		0.8 (0.61-1.06)	0.75 (0.47-1.18)		1.09 (0.74-1.61)	
		Other area	0.69 (0.69-1.45)		0.59 (0.26-1.36)	0.68 (0.18-2.6)		2.11 (0.73-6.05)	
	**Educational attainment (reference=secondary school and below)**								
		Some college	1.03 (1.03-1.34)		1.05 (0.79-1.39)	1.37 (0.87-2.15)		0.92 (0.62-1.35)	
		Completed college	1.1 (1.1-1.38)		1.09 (0.86-1.4)	1.23 (0.81-1.85)		0.83 (0.6-1.16)	
		Other	1.44 (1.44-2.19)		1.11 (0.71-1.75)	1.07 (0.51-2.26)		0.7 (0.37-1.3)	
	**Employment status (reference=not formally employed)**								
		Formally employed	1.16 (1.16-1.4)		0.99 (0.81-1.22)	1.07 (0.78-1.46)		1.02 (0.78-1.34)	
	**Economic situation (reference=stayed the same)**								
		Got worse	0.87 (0.87-1.04)		1.02 (0.84-1.24)	*1.39* ^b^ *(1.02-1.89)*		*1.75* ^e^ *(1.34-2.29)*	
		Got better	1.26 (1.26-1.72)		1.06 (0.75-1.5)	1.18 (0.67-2.11)		1.29 (0.8-2.06)	
	**Cohabitation status (reference=not living with a partner)**								
		Living with partner	1.08 (1.08-1.31)		1.13 (0.91-1.39)	0.82 (0.59-1.14)		0.88 (0.66-1.17)	
	**Children at home**								
		Has children at home	1.13 (1.13-1.45)		1.08 (0.83-1.41)	1.05 (0.69-1.61)		1.01 (0.71-1.42)	
	Gross domestic product per capita (US $, thousands)			*0.99* ^f^ *(0.98-1.00)*	1.01 (0.99-1.03)		1.02 (0.98-1.05)		1.00 (0.98-1.01)
**Supplementary multivariable analysis with sexual orientation variable^g,h^**									
	**Sexual orientation (reference=heterosexual)**								
		Nonheterosexual	*1.35* ^f^ *(1.08-1.69)*		*1.58* ^e^ *(1.24-2.02)*	*1.55* ^b^ *(1.09-2.21)*		0.91 (0.66-1.26)	

^a^aOR: adjusted odds ratio.

^b^*P*<.05.

^c^Values in italics format indicate findings where *P*<.05 (statistical significance).

^d^Not available.

^e^*P*<.001.

^f^*P*<.01.

^g^Please note that sexual orientation was not measured systematically in several countries and therefore was not included as a variable in the main analysis. However, it was an important factor; therefore, we have chosen to display the role of sexual orientation in telemedicine use, adjusted for all of the above variables in a separate model.

^h^Telemedicine use before COVID-19: n=1657, telemedicine use during COVID-19: n=1467, switched to telemedicine during COVID-19: n=881, and poor experience with telemedicine during COVID-19: n=870.

Third, for those who never used telemedicine prior to COVID-19 but switched to using telemedicine during COVID-19, participants who perceived that they were worse off financially were more likely to have switched to telemedicine during COVID-19 (aOR 1.39, 95% CI 1.02-1.89). For those who reported a fair or poor experience of telemedicine, participants who perceived that they were worse off financially were more likely to report doing so (aOR 1.75, 95% CI 1.34-2.29). When sexual orientation was included in the model, we noted that nonheterosexual individuals were more likely to ever use telemedicine prior to COVID-19 (aOR 1.35, 95% CI 1.08-1.69), more likely to have used telemedicine during COVID-19 (aOR 1.58, 95% CI 1.24-2.02), and more likely to have switched to telemedicine during COVID-19 (aOR 1.55, 95% CI 1.09-2.21).

## Discussion

### Principal Findings

This study explored the use of telemedicine during COVID-19 through an analysis of the I-SHARE-2 study that took place amid the lifting of COVID-19 restrictions. Slightly over half the participants we surveyed had never used telemedicine prior to the COVID-19 pandemic. However, this proportion increased during the pandemic, with about a quarter of those who never used telemedicine before switching to the use of telemedicine. About a third of participants reported a fair or poor telemedicine experience during this time period. This study extends the literature through an analysis of multiple countries and provides insight into the use of different telemedicine modalities and the factors associated with them during the COVID-19 pandemic. In this section, we discuss these findings considering the available evidence and suggest implications of our data for policy and future research.

### Comparison to Prior Work

Our study found that about less than half of the participants had ever used telemedicine services prior to the COVID-19 pandemic. This is lower than estimates published by Ipsos in 2018, which estimated that globally, only 10% of individuals ever reported using telemedicine. Nevertheless, such estimates vary widely across contexts, with Saudi Arabia (31%), India (27%), China (24%), Malaysia (15%), and the United States (15%) being the highest adopters in 2018 [20].

Furthermore, we found that those who were older were less likely to have used telemedicine prior to the pandemic. The finding on the association between older age and lower use of telemedicine has been supported in past studies around the world due to barriers that older patients face in terms of accessing these technological platforms [21-23]. It is worth noting that we did not find an association between age and switching to the use of telemedicine during the pandemic. The findings on this association are mixed. Some contexts find no difference in age and use rates for telemedicine [23]. However, this contrasts with other studies, which found that older adults were less likely to take up telemedicine services compared to younger people during the pandemic [24-26]. Nevertheless, the negative correlation between increasing age and low telemedicine use is not universal and may differ across country settings, disease areas, and types of telemedicine platforms (eg, video, audio, or text-based) [27]. Further nuanced research on different older adult populations, as well as modes of using telehealth, is warranted.

Our study also indicated that individuals who identified as sexual minorities were more likely to have ever used telemedicine services. They were also more likely to have used telemedicine services during the pandemic. These results are unsurprising, given that traditional facility-based health services have often been associated with stigma and suboptimal care for sexual minorities [19,28] and that digital health interventions targeted at sexual minorities have been expanding across most settings [29]. There has also been evidence to show that services for sexual minorities had successfully pivoted to provide telehealth services during the pandemic, thus offering targeted interventions for this population that might have increased their access to telemedicine services.

The adoption of telemedicine during the pandemic, among individuals who previously never used telemedicine, was also influenced by economic circumstances. Our results indicated that those who perceived themselves as financially worse off during the pandemic were more likely to switch into digital care but at the same time also report fair or poor telemedicine experiences. This substantial proportion of individuals reporting fair or poor experiences with telemedicine use is noteworthy, given that a systematic review on telemedicine use in low- to middle-income countries found high patient satisfaction with such services [30]. Another systematic review found high satisfaction across multiple medical disciplines [31]. Ultimately, our data may reflect how some individuals who may have competing financial demands may not have been able to leverage technology effectively to enjoy telemedicine services compared to others who were more financially stable or better off during the pandemic.

Audio-based telemedicine services were the most used platform across both high- and low-income countries in our sample. We noted that more participants in high-income countries switched to the use of video-based telemedicine services (before: n=36, 9.1% and during: n=116, 20.5%) compared to those in low-income countries (before: n=113, 13.8% and after: n=81, 13.9%). We also note that chat-based services were the second most popular platform for the use of telemedicine services in low-income countries in our sample. These findings may reflect disparities in technological and telecommunication infrastructures between high- and low-income settings, which may shape access to telemedicine. Studies have found that chat-based platforms may be preferred over video-based platforms in low- to middle-income countries due to the reduced need for data bandwidth and stability of communications [30].

We are mindful of several limitations of this study. This study relies on self-reported data, which may be subject to recall bias and social desirability bias. Participants might not accurately remember their use of telemedicine or may report what they perceive as socially acceptable answers. There is also the possibility of nonresponse bias of those individuals who did not participate in the survey and might have different experiences or views about telemedicine compared to those who participated. Moreover, the survey was conducted on the web, which might result in a selection bias where respondents who are more technology-savvy or have better access to digital devices or internet services were recruited. Such participants may already have better experiences of telemedicine given their technological savvy. The cross-sectional design also constitutes a limitation to establish causality. Additionally, the study methodology is basically quantitative, which may not capture the nuanced experiences, perceptions, and challenges faced by users of telemedicine. As only 8 countries have been included, the findings may not be generalizable to all populations and may not represent the global diversity in health care systems, technological infrastructure, and cultural attitudes toward telemedicine. Moreover, the study groups various forms of telemedicine (audio, video, text, and chat) into a single category. The experiences and effectiveness of these different modalities can vary greatly, and lumping them together might oversimplify the analysis.

### Conclusions

Most of the participants had never used telemedicine before the pandemic, and a significant proportion did not use it either following the introduction of COVID-19 measurements. Only a quarter of the participants who never used it before switched into it during the pandemic. However, about one-third of the participants reported fair or poor experiences using it during COVID-19. Telemedicine has emerged as a critical tool for expanding access to health care services, particularly in the field of SRH. The COVID-19 pandemic has accelerated its adoption, highlighting the importance of telemedicine during crises, emergencies, or natural disasters. Understanding all the factors that influence the implementation of digital consultations, barriers and facilitators could help not only health care professionals but also policy makers and stakeholders to tailor strategies that improve its accessibility and effectiveness in delivering proper care. Finally, the adoption and implementation of telemedicine may be cost-saving but might also incur costs that vary greatly across different countries and contexts due to diverse health system configurations and digital and telecommunication infrastructures [32]. Future work should therefore consider how telemedicine services can be used to deliver care to people in a cost-effective manner, especially across high-income and low- to middle-income settings.

## Data Availability

The datasets generated and analyzed during this study are available from the corresponding author on reasonable request.

## Appendix

Multimedia Appendix 1 Supplementary data containing the survey instrument as well as figures summarizing telemedicine modalities used before and during COVID-19 measures by country among International Sexual Health and Reproductive Health-2 study participants in 8 countries, April 2021 to July 2022.

## Abbreviations

aOR: adjusted odds ratio
GDP: gross domestic product
I-SHARE: International Sexual Health and Reproductive Health
SRH: sexual and reproductive health

## References

[1] (2010). Telemedicine: opportunities and developments in Member States: report on the second global survey on eHealth. World Health Organization.

[2] Sood S, Mbarika V, Jugoo S, Dookhy R, Doarn CR, Prakash N, Merrell RC (2007). What is telemedicine? A collection of 104 peer-reviewed perspectives and theoretical underpinnings. Telemed J E Health.

[3] Haleem A, Javaid M, Singh RP, Suman R (2021). Telemedicine for healthcare: capabilities, features, barriers, and applications. Sens Int.

[4] (2022). Universal access to sexual and reproductive health. World Health Organization.

[5] Monaghesh E, Hajizadeh A (2020). The role of telehealth during COVID-19 outbreak: a systematic review based on current evidence. BMC Public Health.

[6] (2024). Telemedicine—statistics and facts. Statista.

[7] Nanda K, Lebetkin E, Steiner MJ, Yacobson I, Dorflinger LJ (2020). Contraception in the era of COVID-19. Glob Health Sci Pract.

[8] Qaderi K, Khodavirdilou R, Kalhor M, Behbahani BM, Keshavarz M, Bashtian MH, Dabir M, Irani M, Manouchehri E, Farahani MF, Mallah MA, Shamsabadi A (2023). Abortion services during the COVID-19 pandemic: a systematic review. Reprod Health.

[9] Bittleston H, Goller JL, Temple-Smith M, Hocking JS, Coombe J (2022). Telehealth for sexual and reproductive health issues: a qualitative study of experiences of accessing care during COVID-19. Sex Health.

[10] Cheng Y, Boerma C, Peck L, Botfield JR, Estoesta J, McGeechan K (2021). Telehealth sexual and reproductive health care during the COVID-19 pandemic. Med J Aust.

[11] VanBenschoten H, Kuganantham H, Larsson EC, Endler M, Thorson A, Gemzell-Danielsson K, Hanson C, Ganatra B, Ali M, Cleeve A (2022). Impact of the COVID-19 pandemic on access to and utilisation of services for sexual and reproductive health: a scoping review. BMJ Glob Health.

[12] Doraiswamy S, Abraham A, Mamtani R, Cheema S (2020). Use of telehealth during the COVID-19 pandemic: scoping review. J Med Internet Res.

[13] Elson E C, Oermann C, Duehlmeyer S, Bledsoe S (2020). Use of telemedicine to provide clinical pharmacy services during the SARS-CoV-2 pandemic. Am J Health Syst Pharm.

[14] Perrin PB, Pierce BS, Elliott TR (2020). COVID-19 and telemedicine: a revolution in healthcare delivery is at hand. Health Sci Rep.

[15] Arafat MY, Zaman S, Hawlader MDH (2021). Telemedicine improves mental health in COVID-19 pandemic. J Glob Health.

[16] Michielsen K, Larrson EC, Kågesten A, Erausquin JT, Griffin S, van de Velde S, Tucker JD (2021). International Sexual Health and Reproductive Health (I-SHARE) survey during COVID-19: study protocol for online national surveys and global comparative analyses. Sex Transm Infect.

[17] Erausquin J, Tan RKJ, Uhlich M, Francis JM, Kumar N, Campbell L, Zhang WH, Hlatshwako TG, Kosana P, Shah S, Brenner EM, Remmerie L, Mussa A, Klapilova K, Mark K, Perotta G, Gabster A, Wouters E, Burns S, Hendriks J, Hensel DJ, Shamu S, Strizzi JM, Esho T, Morroni C, Eleuteri S, Sahril N, Low WY, Plasilova L, Lazdane G, Marks M, Olumide A, Abdelhamed A, López Gómez A, Michielsen K, Moreau C, Tucker JD (2022). The International Sexual Health and Reproductive Health Survey (I-SHARE-1): a multi-country analysis of adults from 30 countries prior to and during the initial COVID-19 wave. Clin Infect Dis.

[18] Michielsen K, Campbell L, de la Torre FF (2022). The impact of COVID-19 on sexual and reproductive health in Eastern Europe and Central Asia. UNFPA.

[19] Tan RKJ, Kaur N, Kumar PA, Tay E, Leong A, Chen MI, Wong CS (2020). Clinics as spaces of costly disclosure: HIV/STI testing and anticipated stigma among gay, bisexual and queer men. Cult Health Sex.

[20] (2018). Global views on healthcare in 2018. Ipsos.

[21] Pasquinelli MM, Patel D, Nguyen R, Fathi J, Khan M, Fernandez K, Bhatia Y, Corbridge S, Cadman K, Harmon V, Trosman J, Weldon C, Pappalardo AA, Nyenhuis SM (2022). Age-based disparities in telehealth use in an urban, underserved population in cancer and pulmonary clinics: a need for policy change. J Am Assoc Nurse Pract.

[22] Braswell M, Wally MK, Kempton LB, Seymour RB, Hsu JR, Karunakar M, Afetse KE, Bailey Gi, Bosse M, Brownrigg M, Cuadra M, Dixon A, Girardi C, Grochowski E, Hysong A, Jolissaint J, Macknet D, Mayberry RM, Moody P, Peterson K, Phelps KD, Pollock H, Posey SL, Reid R, Roe K, Scannell B, Sims S, Stanley A, Wohler AD (2021). Age and socioeconomic status affect access to telemedicine at an urban level 1 trauma center. OTA Int.

[23] Hayrapetian L, Zepp M, Rao S, Hennessey M, Houle M, Atienza M, Belfaqeeh OA, Dharia I, Khan A, Borum ML (2021). Expanding telehealth options during the COVID pandemic eliminated racial and age disparities in electronic communication by inflammatory bowel disease patients. J Natl Med Assoc.

[24] Miyawaki A, Tabuchi T, Ong MK, Tsugawa Y (2021). Age and social disparities in the use of telemedicine during the COVID-19 pandemic in Japan: cross-sectional study. J Med Internet Res.

[25] Choi NG, DiNitto DM, Marti CN, Choi BY (2022). Telehealth use among older adults during COVID-19: associations with sociodemographic and health characteristics, technology device ownership, and technology learning. J Appl Gerontol.

[26] Eberly LA, Kallan MJ, Julien HM, Haynes N, Khatana SAM, Nathan AS, Snider C, Chokshi NP, Eneanya ND, Takvorian SU, Anastos-Wallen R, Chaiyachati K, Ambrose M, O'Quinn R, Seigerman M, Goldberg LR, Leri D, Choi K, Gitelman Y, Kolansky DM, Cappola TP, Ferrari VA, Hanson CW, Deleener ME, Adusumalli S (2020). Patient characteristics associated with telemedicine access for primary and specialty ambulatory care during the COVID-19 pandemic. JAMA Netw Open.

[27] Friedman EE, Devlin SA, Gilson SF, Ridgway JP (2022). Age and racial disparities in telehealth use among people with HIV during the COVID-19 pandemic. AIDS Behav.

[28] O'Hara CA, Foon XL, Ng JC, Wong CS, Wang FY, Tan CY, Cheah YT, Griva K, Yoong JS, Tan RK (2023). Lesbian, gay, bisexual, transgender, queer and intersex (LGBTQI+) healthcare in Singapore: perspectives of non-governmental organisations and clinical year medical students. Med Educ Online.

[29] Waad A (2019). Caring for Our Community: telehealth interventions as a promising practice for addressing population health disparities of LGBTQ+ communities in health care settings. Dela J Public Health.

[30] Tiwari BB, Kulkarni A, Zhang H, Khan MM, Zhang DS (2023). Utilization of telehealth services in low- and middle-income countries amid the COVID-19 pandemic: a narrative summary. Glob Health Action.

[31] Pogorzelska K, Chlabicz S (2022). Patient satisfaction with telemedicine during the COVID-19 pandemic—a systematic review. Int J Environ Res Public Health.

[32] Snoswell CL, Taylor ML, Comans TA, Smith AC, Gray LC, Caffery LJ (2020). Determining if telehealth can reduce health system costs: scoping review. J Med Internet Res.

