# Supplementary material for: Telemedicine Use During the COVID-19 Pandemic in 8 Countries From the International Sexual Health and Reproductive Health Consortium: Web-Based Cross-Sectional Survey Study
Source: J Med Internet Res. 2025 Mar 4;27:e60369. doi: 10.2196/60369 (PMC11920653; doi:10.2196/60369)
Supplement: Multimedia Appendix 1 [file jmir_v27i1e60369_app1.docx]

**Table S1. Survey instrument with variables used in this study for the second wave of the International Sexual Health And REproductive Health (I-SHARE) consortium study**

| **Question** | **Response (Please refer to choice sheet following this survey instrument for more details)** |
| --- | --- |
| How old are you? |  |
| **Socio-demographics** |  |
| What sex were you assigned at birth? | Woman  Man  Other |
| Which of the following do you identify as? | Woman  Man  Both  Neither  Other |
| What best describes the area where you live? | City  Suburb of city  Town  Remote/rural area  Other |
| What best describes your relationship status? | Single  Currently in a relationship but not living together  Currently in a relationship and living together  Widowed  Divorced or separated  Other |
| Do you have children? |  |
| What is your highest degree of schooling? | No formal education  Some primary school  Completed primary school  Some secondary school  Completed secondary school  Some college or university  Completed college or university  Other |
| If other, please specify |  |
| Since the introduction of the COVID measures, the economic situation of many households has changed. Has this been the case for you? | Yes, the economic situation of my household became worse  No, the economic situation of my household stayed the same  Yes, the economic situation of my household improved |
| What was your employment status the month before the introduction of the COVID-19 measures on [MEASURES DATE]? (multiple answers possible) | Employed and received a salary  Self-employed / Business owner  Unemployed  Informal / Piecemeal work  Retired / Pensioned  Student  Other |
| In the last three months, is your employment status different when compared to before the introduction of the COVID-19 measures on [MEASURES DATE]? | No change: I continue doing the same work and going to the usual job site  I keep doing the same work, but from home  I keep doing the same work, but partly from home  I am employed and paid but unable to attend or do work  I work on reduced time  I lost my job/work/business  I am temporarily unemployed  I changed work/jobs  Other |
| If other, please specify |  |
|  |  |
| **Telemedicine** |  |
| Before the initial introduction of COVID-19 measures, had you ever consulted a health care provider by phone, chat or email? | Never  Rarely  Sometimes  Often  Always |
| Tick the options that you used | Audio service such as telephone  Visual service such as video chat  Text messages  Internet chat messages  Other |
| Following the introduction of the COVID-19 measures, have you consulted a health care provider by phone, chat or email? | No  Yes  Not applicable (did not need health care) |
| Tick the options that you used | Audio service such as telephone  Visual service such as video chat  Text messages  Internet chat messages  Other |
| How would you rate your satisfaction with the health care received when communicating with healthcare providers via phone/chat/email since COVID-19? | Excellent  Good  Fair  Poor |

**Figure S1. Telemedicine modalities used before COVID-19 measures by country among I-SHARE-2 study participants in eight countries, April 2021 to July 2022 (n=2857)**

**Figure S2. Telemedicine modalities used during COVID-19 by country among I-SHARE-2 study participants in eight countries, April 2021 to July 2022 (n=2459)**
